# Prevalence and perceived health effect of alcohol use among male undergraduate students in Owerri, South-East Nigeria: a descriptive cross-sectional study

**DOI:** 10.1186/1471-2458-11-118

**Published:** 2011-02-18

**Authors:** Ebirim IC Chikere, Morakinyo O Mayowa

**Affiliations:** 1 Department of Public Health Technology, School of Health Technology, Federal University of Technology, Owerri, South-East Nigeria

## Abstract

**Background:**

Alcohol use during adolescence and young adulthood remains a prominent public health problem. Despite growing problems of global alcohol abuse, accurate information on the prevalence and pattern of use in Nigeria remain sparse. This study examines the prevalence and perceived health effects of alcohol use among undergraduate students in Owerri, Nigeria.

**Method:**

The prevalence and perceived health effects of alcohol was estimated for 482 male undergraduates of four higher institutions in Owerri, South-East Nigeria between October 2008 and March 2009. Information was obtained using a semi-structured, self-administered questionnaire.

**Result:**

The mean age of the students was 24.7 years. Majority of the respondents confirmed they were current users of alcohol given a prevalence of 78.4%, with twenty-seven percent of them being heavy drinkers (≥4 drinks per day). Reasons given by respondents for alcohol drinking include: makes them feel high (24.4%); makes them belong to the group of "most happening guys" on campus (6.6%); makes them feel relaxed (52.6%) while (16.4%) drinks it because their best friends do. Perceived health impacts of alcohol use among current users include: it enhances pleasure during moment of sex (51.1%), causes drowsiness and weakness (63.8%), may precipitate defective memory and impaired perception (64.3%) and serves as risk factor for most chronic diseases (68.5%).

**Conclusion:**

High prevalence of alcohol use was established among study groups. Evaluation of full-scale community-level intervention, including community mobilisation and media advocacy aimed at supporting changes in policies on drinking, access and sales of alcohol to young people, could be helpful in reducing the trend.

## Background

Alcohol consumption has occurred for thousands of years. In many parts of the world, drinking alcoholic beverages is a common feature of social gatherings [1]. While alcohol use is deeply embedded in many societies, recent years have seen changes in drinking patterns across the globe: rates of consumption, drinking to excess among the general population and heavy episodic drinking among young people are on the rise in many countries. Nevertheless, the consumption of alcohol carries a risk of adverse health and social consequences related to its intoxicating, toxic and dependence-producing properties [1].

Alcohol consumption has health and social consequences via intoxication (drunkenness), dependence (habitual, compulsive and long-term drinking), and other biochemical effects. In addition to chronic diseases that may affect drinkers after many years of heavy use, alcohol contributes to traumatic outcomes that kill or disable one at a relatively young age, resulting in the loss of many years of life to death or disability. Alcohol is estimated to cause about 20-30% worldwide disease of oesophageal cancer, liver cancer, cirrhosis of the liver, homicide, epilepsy, and motor vehicle accidents [1].

Globally, alcohol consumption has increased in recent decades, with all or most of that increase in developing countries. World-wide, five percent of all deaths of people between the ages of 5 and 29 in 1990 were attributable to alcohol use. The Global Burden of Disease Study found that alcohol was responsible in 1990 for 3.5 percent of all disability-adjusted life years (more than tobacco or illegal drugs). While adverse health outcomes from long-term chronic alcohol use may not cause death or disability until late in life, acute health consequences of alcohol use, including intentional and unintentional injuries, are far more common among younger people [2].

In Nigeria, psychoactive substance misuse especially alcohol has for many years been an issue of increasing health and social importance. This is especially so for the critical adolescent period marked by several changes including the psychological phenomenon of experimentation. Studies carried out in the last decade in Nigeria have identified adolescents as a major group involved in the use of alcohol. This study describes the prevalence of alcohol use among male undergraduate students in Owerri, South-East, Nigeria. It also documents the perceived health effects of alcohol use among the study group.

## Methods

From October 2008 to March 2009 a descriptive cross-sectional survey was conducted among Undergraduate students from four tertiary institutions in Owerri. These institutions include Federal University of Technology (FUTO), Imo State University (IMSU), Alvan Ikoku Federal College of Education (AIFCE) and Federal Polytechnic Nekede (FPN), all in Owerri South-east, Nigeria.

Owerri is a city in South-Eastern Nigeria. It is the capital of Imo State and is set in the heart of the Igbo land. Owerri consists of three LGAs (Owerri Municipal, Owerri North and Owerri West). It currently has a population of about 400,000 and is approximately 40 square miles (100 km^2^) in area. It is bordered by the Otamiri River to the east and the Nworie River to the south. It occupies the area lying between coordinates 5.484°N and 7.035°E.

A multi stage sampling design was used to select participants. Interviews using structured questionnaires were conducted with randomly selected respondents in six faculties from four institutions. The questions focused on various sub-themes like socio-demographic information, prevalence of alcohol use, reasons for alcohol use, number of bottles drank per day and perceived impact of alcohol use among the respondents. A copy of the questionnaire containing the variables of interest is contained in additional file 1.

Questionnaire was prepared in English and was self-administered. It was administered after explaining the purpose of the study and criteria used in selecting each respondent. Permission to conduct the survey was requested and obtained from the university ethical review board. Informed verbal and written consent was obtained from participants. Confidentiality of information was maintained throughout the study.

The data collected was manually sorted out, edited and coded. It was thereafter imputed into the computer for analysis using SPSS version 15.0 statistical package. Frequency tables were generated for demographic characteristics of the respondents. Qualitative variables were summarized by proportions. Students who drank four bottles or more per day were classified as heavy drinkers while those dinking less were classified as non-heavy drinkers. Statistical significance for association was tested using chi-square, with p-value less than 0.05 considered statistically significant. The commonest alcoholic drink among the study population was 'Star'. A bottle of Star is 60centiliters in volume with an alcohol content of 5.1% per volume.

## Results

A total of 482 records of male tertiary undergraduates from four higher institutions in Owerri were available and analysed. Majority of the respondents, 42.5% compared to 16.6% who were in the 16 - 20 years age group. Most of the respondents (79.0%) were unmarried whereas only 21.0% were married as shown in Table 1. The sample studied includes 24.5% of IMSU students, 23.9% of FUTO students, 26.6% of FPN students and 25.1% of AIFCE students. The percentage of individuals who were Christians is higher than those that were Muslims.

**Table 1:** Demographic Characteristics of Respondents

Age Group	Frequency (N = 482)	Percentage
16 - 20	80	16.6
21 - 25	197	40.9
26 & above	205	42.5

**Marital Status**		

Married	101	21.0
Single	381	79.0

**Institution**		

IMSU	118	24.5
FUTO	115	23.9
FED POLYTECH	128	26.6
AICE	121	25.1

**Faculty**		

Engineering	143	29.7
Medical	51	10.6
Business College	122	25.3
Science	94	19.5
Law School	3	0.6
Humanities	66	13.7
Others	3	0.6

**Religion**		

Christian	460	95.4
Islam	22	4.6

**Year of Study**		

1^st ^Year	53	11.0
2^nd ^Year	90	18.7
3^rd ^Year	151	31.3
4^th ^Year	188	39.0

**Ethnicity**		

Igbo	444	92.1
Hausa	22	4.6
Igbo	16	3.3

The prevalence of alcohol consumption among respondents vis-a-vis their demographic information is shown in Table 2. Prevalence of alcohol use is high with 78.4% among all respondents and 92.2% among students aged 26 years and above (p-value < 0.001). The percentage of single students that use alcoholic drink was significantly higher than those that are married (p-value < 0.001).

**Table 2:** Prevalence of Alcohol Use among Respondents

Category	Total Number (N = 482)	Alcohol users (N = 378)	Prevalence (%)	P value
**Age (years)**				
16 - 20	80	56	70.0%	< 0.001
21 - 25	197	133	67.5%	
≥26	205	189	92.2%	

**Marital Status**				

Married	101	92	91.1%	
Single	381	286	75.1%	< 0.001

**Religion**				

Christian	460	367	79.8%	
Islam	22	11	50.0%	0.001

**Year of Study**				

1^st ^Year	53	27	50.9%	
2^nd ^Year	90	77	85.6%	< 0.001
3^rd ^Year	151	130	86.1%	
4^th ^Year	188	144	76.6%	

**Ethnicity**				

Igbo	444	351	79.1%	
Hausa	22	11	50.0%	0.001
Yoruba	16	16	100.0%	

Table 3 present reasons why respondents use alcohol and other important parameter. About 24.4% of the respondents said it makes them feel high (on top of the world); 6.6% claimed that it makes them belong to the group of "most happening guys" on campus; 52.6% said it makes them feel relaxed, helps in cooling off stress; while the remaining 16.4% respondents said they indulged in the act of using alcohol because their best friends drink it.

**Table 3:** Distribution of Reasons for Alcohol Use and related parameters among Respondents

Variables	Frequency (N = 378)	Percentage
**Reason for alcohol use**		

It makes me to belong	25	6.6
All my friends are alcoholics	62	16.4
It makes me feel high	92	24.3
Other reasons	199	52.6

**Age at alcohol Initiation**		

11 - 15	44	11.6
16 - 20	170	45.0
21 - 25	144	38.1
26 & above	20	5.3

**Source of alcohol**		

School cafe	61	46.1
Night club	90	23.8
Anywhere	147	38.9
Roadside joints	52	13.8
Others	28	7.8

**Introduction to alcohol Use**		

Family member	101	26.7
Friend	177	46.8
Fellow student	9	2.4
Neighbour	30	7.9
Others	61	16.1

**No of bottles Per Day**		

1 - 3	277	73.3
4 -6	73	19.3
7-10	25	6.6
11 & above	3	0.8

**Intent to Quit**		

Yes	181	47.9
No	197	52.1

**Reason for Intent**		

Religious reasons	14	7.8
Advice from superior	14	7.7
Medical reasons	113	62.4
Other	40	22.1

Table 4 documents the impact/effects of alcohol use on the respondents. The table shows that 45.5% of the 378 alcohol users admitted that it makes them feel bad, while 55.5% said it gives them good feeling. Majority said it enhances pleasure during moment of sex; 46.3% usually have residual depressive feeling of remorse hours after use; while 63.8% reported it causes drowsiness, weakness, hangovers, dangerous driving speed and may lead to accident. The respondents when classified according to the number of bottles of alcohol drunk per day revealed that 101 (26.7%) of 378 respondents that have ever used alcohol were heavy drinkers.

**Table 4:** Impacts/Effects of Alcohol use Among Respondents

Effect	Frequency (N = 378)	Percentage
**Gives good feeling**		

Agree	206	54.5
Disagree	172	45.5

**Serves as risk factor for most diseases**		

Agree	259	68.5
Disagree	119	31.5

**It enhances moment of sex**		

Agree	193	51.1
Disagree	185	38.9

**It gives sense of warmth**		

Agree	89	23.5
Disagree	289	76.5

**Completes social gathering and celebration**		

Agree	265	70.1
Disagree	113	29.9

**Used for checking weight**		

Agree	92	24.3
Disagree	286	75.7

**Leads to strained relationship**		

Agree	97	25.7
Disagree	281	74.3

**Cause residual depressive feeling of remorse**		

Agree	175	46.3
Disagree	203	53.7

**Causes absenteeism and poor performance in school**		

Agree	220	58.2
Disagree	158	41.8

**It precipitates mental symptoms**		

Agree	243	64.3
Disagree	135	35.7

**Cause drowsiness, weakness, hangover and accident**		

Agree	241	63.8
Disagree	137	36.2

## Discussion

Valid information on prevalence of alcohol is important input for public health policy. The results of this investigation indicate that alcohol consumption has a prevalence of 78.4% among the respondents. Survey and anecdotal data from countries around the globe suggest that a culture of sporadic heavy or "binge" drinking among young people may be spreading from the developed to the developing countries [3].

A number of school and college surveys in Nigeria have found alcohol use to be common among students, with many drinking students having had their first drink in family settings [4]. In June 1988 a questionnaire survey of 636 undergraduate students at the University of Ilorin in Kwara State found that 77% reported lifetime alcohol use (81 per cent of men and 68 per cent of women) [5]. In response to a 1988 survey of 1,041 senior secondary school students in Ilorin, 12% reported current use of alcohol [6].

Findings from this study show that majority of the respondents were initiated into the use of alcohol at a tender age of 16 to 20 years. Previous studies have shown that age of initiation of alcohol use is important. Research in the US has found that the earlier the age at which people begin drinking, the more likely they are to become alcohol dependent later in life [7]. Those who begin drinking in their teenage years are also more likely to experience alcohol-related unintentional injuries (such as motor vehicle injuries, falls, burns, drowning) than those who begin drinking at a later age [8]. Adverse effects of early onset of drinking may be shorter term as well: prospective research has found a younger age of initiation to be strongly related to a higher level of alcohol misuse at ages 17 and 18 [9].

Moreover, certain individuals (26.7%) vividly reported that they were introduced to alcohol by their family members. This is consistent with a collaborative report from WHO's European Regional Office which estimated that 4.5 million young people lived in families adversely affected by alcohol [10]. Problems for the young people in such homes may include instability or collapse of marriages and family structures, increased risk of physical or sexual abuse, neglect, and strain on family finances. Such family problems may in turn put young people at greater risk of developing anti-social behaviours, emotional problems and problems in the school environment [11].

Documentation of quantity of alcohol consumed revealed that 26.7% were heavy drinkers and at risk of most of the health complication associated with alcohol consumption. Harmful use of alcohol encompasses several aspects of drinking; one is the volume drunk over time. The strongest drinking-related predictor of many chronic illnesses is the cumulated amount of alcohol consumed over a period of a year. Also, the risks of intentional and unintentional injuries and of transmission of certain infectious diseases are predicted by the pattern of drinking, occasional or regular drinking, drinking to intoxication and the drink context.

The range of adverse physical consequences stemming from heavy use of alcohol on a single occasion is well documented. The most obvious of these is alcohol poisoning, which although relatively rare is often emblematic of young drinkers' inexperience with alcohol. Alcohol may have a more immediate and severe effect on young people because their muscle mass is smaller than that of adults (WHO, 2009).

While evidence is inconclusive regarding the direct impact of alcohol use on the physical development of young people, there are indications that heavy alcohol use at a young age is predictive of a range of psychological and physical problems. Protracted and continuous abuse of alcohol may be predictive of more severe health problems in general for boys and girls [12]. Alcohol may cause physical harm to children, although the evidence remains preliminary. Studies in laboratory animals have found that high doses of alcohol may delay the onset of puberty, retard bone growth and result in weaker bones [13-15].

Surveys of young people in European countries have looked at a wide range of behavioural consequences of alcohol use. These include individual problems, defined by self-reports on young people's reduced performance at school or at work, damage to objects or clothing, loss of money or other valuable items, and accident or injury as a result of alcohol use. Relationship problems cover self-reported quarrels or arguments, and problems in relationships with friends, teachers or parents as a result of drinking alcohol. Young people also reported on whether they had engaged in unwanted sexual experiences or unprotected sex. Finally, delinquency problems included self-reports of alcohol-related scuffles or fights, victimisation by robbery, or trouble with the police, as well as driving a motorcycle or a car under the influence of alcohol.

When related to their health status, 68.5% accepted that it forms high risk factor for most health problems like ling cancer, liver, sexually transmitted disease, HIV/AIDS, low birth weight in women, stroke and sudden death.

Even though majority (83.4%) accepted the establishment of awareness programmes in their institution on alcohol abuse with seminars, workshops, crusades and conferences held at their school premises, these actions were still not enough to combat the use of alcohol and in the institutions. More effort should be put to strengthen these existing programmes and new line of action developed that can help combat this epidemic.

Among the shortcomings of this study is the fact that there were no previous validated measure for alcohol consumption and alcohol related problems, thus making comparison difficult.

## Conclusions

In conclusion, the prevalence of alcohol consumption (78.4%) is quite high among undergraduates in Owerri and 26.7% of them are at risk of health complications of alcohol consumption. A lot of factors such as peer group pressure, influence of family members, role model, and wrong perception portrayed in advertisement contribute immensely to its use. These factors if checked on time will reduce the prevalence of alcohol consumption among undergraduates and the country at large.

Health implications have proved to be a good reason for non-alcohol use. This means that health education in tertiary institutions will to a reasonable extent reduce the prevalence. Also, since our media stations (especially foreign stations) remain the major media for alcohol advertisement, these media can serve to campaign against their use in the society.

Though, alcohol use is voluntary and is the right of an individual to take alcohol or not, it is equally important and advisable for the government to enforce ban on the sale of alcohol to adolescence so as to protect the health of the future work force of the nation. The government whose ultimate aim is to see to the health of its citizens should allocate adequate resources for campaigns against alcohol use, just as HIV/AIDS awareness has received adequate attention on the part of government.

Numerous evaluation research studies have found that changing certain public policies results in significant effects both on young people's behaviour and on negative outcomes of alcohol consumption. Evaluation of full-scale community-level intervention, including community mobilisation and media advocacy aimed at supporting changes in policies on drinking and driving, access and sales of alcohol to young people, have shown very promising results.

## Competing interests

The authors declare that they have no competing interests.

## Authors' contributions

CICE conceived the study, designed the questionnaire, performed the statistical analysis, and also contributed in drafting of the manuscript.

MOM participated in drafting and critical review of the manuscript.

## Authors Information

**C. I. C. E**. PhD Epidemiology and Disease Control Technology (In-view), M.Sc Epidemiology and Medical Statistics. Lecturer, Public Health Technology Department, Federal University of Technology Owerri, Nigeria.

**M. O. M**. PhD Environmental Management & Toxicology (In-view), MPH Environmental Health. Lecturer, Public Health Technology Department, Federal University of Technology Owerri, Nigeria.

## Pre-publication history

The pre-publication history for this paper can be accessed here:

http://www.biomedcentral.com/1471-2458/11/118/prepub

## Supplementary Material

Additional file 1 **Questionnaire on Prevalence and Perceived Health Effect of Alcohol Use among Male Undergraduate Students in Owerri, South-East Nigeria**. It is a semi-structured questionnaire containing questions on the following sections; demographic characteristics, prevalence of alcohol use, knowledge of health effects of alcohol abuse and perceived health effects of alcohol abuse. Click here for file
