# Supplementary material for: Prevalence and perceived health effect of alcohol use among male undergraduate students in Owerri, South-East Nigeria: a descriptive cross-sectional study
Source: BMC Public Health. 2011 Feb 18;11:118. doi: 10.1186/1471-2458-11-118 (PMC3049753; doi:10.1186/1471-2458-11-118)
Supplement: Additional file 1 — Questionnaire on Prevalence and Perceived Health Effect of Alcohol Use among Male Undergraduate Students in Owerri, South-East Nigeria. It is a semi-structured questionnaire containing questions on the following sections; demographic characteristics, prevalence of alcohol use, knowledge of health effects of alcohol abuse and perceived health effects of alcohol abuse. [file 1471-2458-11-118-S1.DOC]

**ADDITIONAL FILE 1**

**QUESTIONNAIRE ON PREVALENCE AND PERCEIVED HEALTH EFFECT OF ALCOHOL USE AMONG MALE UNDERGRADUATE STUDENTS IN OWERRI, SOUTH-EAST NIGERIA**

**INTRODUCTION AND CONSENT**

I am conducting a study that has to do with alcohol use in undergraduates and its impact on youths. It is hoped that the result of the finding will help us to know the factors predisposing youths to alcohol abuse and cigarette smoking and finding possible solutions to it.

May I ask you a few questions? It will take only a few moments of your time and I think you will find it interesting. Please do not write your name on the questionnaire as your confidentiality is guaranteed. Thank you for your participation.

For official use

Sign: _____________________

Location: _____________________

Name of institution: _______________________

**SECTION A**

**DEMOGRAPHIC CHARACTERISTICS**

1. Serial number**___________________**

. 2. Age last birthday? ____________ Years

3. Religion (1) Christianity (2) Islam (3) Traditional

(4) Others (please specify) -------------------

4. Ethnicity (1) Yoruba (2) Hausa (3) Igbo (4) Others------

5. Level/year of study_______________________________

6. Faculty ______________________________

**SECTION B**

### PREVALENCE OF ALCOHOL USE

7. Have you ever taken alcoholic drink before? (1) Yes (2) No

8. What was your reason for drinking your first alcohol?_________________________

9. At what age did you first drink alcohol? ______________

10. Who introduced you into alcohol drinking? (1). Family member (2). Friends

(3). Fellow students (4) Neighbors (5) Other specify_____________

11. Do you currently drink alcoholic drinks? (1) Yes (2) No

12. If “No” when did you quit drinking? _____________________

13 Where do you most times get your supply of alcohol? (1)School (2) House (3)Anywhere (4) joints ( 5) club house

14. Do you drink alcohol daily for the past one year? (1).Yes (2). No

15. How many bottles do you usually drink per day? …………..

16. Do any of your parents/ guardian drink alcohol? (1) Yes (2) No

17. Do your relatives/siblings drink in your presence? (1)Yes (2) No

18. Which of the following alcohol beverages have you used?

| S/N | SUBSTANCES | Yes | No |
| --- | --- | --- | --- |
| A | beer |  |  |
| B | stout |  |  |
| C | Red wine |  |  |
| D | Fermented Palm wine |  |  |
| E | Distilled spirit ( rum, gin) |  |  |
| F | Fortified spirit (brandy and barley) |  |  |

### 19. Do you intend to remain an alcohol drinker

(1) Yes (2) No

20. Do you have friends that drink alcohol? (1)Yes (2) No

21. Do your role model drink alcohol? ( 1 ) Yes (2) No

**SECTION C**

**Knowledge of Health Effect of Alcohol Abuse**

22. What health effects of alcohol are you aware of? __________________________

23. Where do you hear about the health effects of alcohol misuse? _____________

24. Is there any current program in your institution to prevent alcohol abuse?

(1) Yes (2) No

25. Which of the following health or health related effects may be associated with the consumption of some of the alcohol beverages?

| Likely associated | Associated | Not associated |
| --- | --- | --- |
|  |  |  |
|  |  |  |
|  |  |  |
|  |  |  |
|  |  |  |
|  |  |  |
|  |  |  |
|  |  |  |
|  |  |  |
|  |  |  |

- (1)Traffic and other accidents
- (2) low birth weight in women
- (3) stroke and sudden death
- (4)HIV and other STIs
- (5)Liver diseases
- (6)Heart diseases
- (7)Respiratory diseases
- (8)Lung cancers and other cancers
- (9)Mental illness
- (10)Lasting effect on the fetus

**SECTION D**

**PERCIEVED HEALTH EFFECT OF ALCOHOL ABUSE**

Instruction; please select the most appropriate option

26. Alcohol consumption gives good feeling

(1) Agree (2) Disagree ( 3) Undecided

27. excessive alcohol intake serves as risk factor for most diseases

(1) Agree (2) Disagree ( 3) Undecided

28. Alcohol intake enhances moment of sex (1) Agree (2) Disagree ( 3) Undecided

29. Alcohol intake gives sense of warmth? (1) Agree (2) Disagree ( 3) Undecided

30. Alcohol intake completes social gathering and celebration (1) Agree (2) Disagree ( 3) Undecided

31. Alcohol intake is often used for checking weight (1) Agree (2) Disagree ( 3) Undecided

32. Alcohol intake leads to strained relationship (1) Agree (2) Disagree ( 3) Undecided

33. Alcohol intake causes depressive feeling of remorse (1) Agree (2) Disagree ( 3) Undecided

34. Alcohol intake causes absenteeism and poor performance in school (1) Agree (2) Disagree ( 3) Undecided

35. Regular alcohol intake precipitates mental symptoms (1) Agree (2) Disagree ( 3) Undecided
